# Subchorionic Placental Cyst in a Woman with Fetal Growth Restriction: A Case Report and Review of the Literature

**DOI:** 10.3390/jcm14196698

**Published:** 2025-09-23

**Authors:** Hyo-Shin Kim, Min-Hye Jang, Yu-Jin Koo, Dae-Hyung Lee

**Affiliations:** 1 Department of Obstetrics and Gynecology, Yeungnam University Medical Center, 170 Hyeonchung-ro, Nam-gu, Daegu 42415, Republic of Korea; 2 Department of Pathology, Yeungnam University Medical Center, Daegu 42415, Republic of Korea

**Keywords:** prenatal ultrasonography, placenta disease, placental insufficiency, pregnancy complications

## Abstract

Subchorionic placental cysts are rare, and their prevalence, etiology, and impact on pregnancy remain unclear. A 40-year-old woman presented at 28^3/7^ weeks of gestation with asymmetric fetal growth restriction. Ultrasonography at 35^3/7^ weeks of gestation revealed a newly detected cystic mass measuring 4 cm in diameter protruding from the fetal surface of the placenta. On pathological examination after delivery, multiple placental infarctions and multifocal perivillous fibrin deposits were diffusely observed throughout the placenta. Through a review of 16 cases of subchorionic placental cysts, including the present case, we found that the majority of cases exhibited abnormal placental pathology suggestive of hypoxia and were associated with unfavorable pregnancy outcomes, such as fetal growth restriction, preterm birth, and an increased risk of cesarean delivery. We suggest that they are associated with unfavorable pregnancy outcomes and may serve as a clinical marker of placental hypoxia.

## 1. Introduction

Subchorionic placental cysts (SPCs) are rare findings, and their incidence and clinical significance are challenging to determine; therefore, only a few cases have been reported in the English scientific literature to date. Not all cysts can be identified prenatally; in addition, not all placentas are routinely submitted for pathological examination, and cystic structures may be irreversibly damaged during delivery [1]. Furthermore, cystic masses on the fetal side of the placental surface have been reported under various names, including “subchorionic cysts,” “placental surface cysts,” “chorionic cysts,” “membranous cysts,” and “subamniotic cysts” [2]. However, recent studies have most commonly referred to these lesions as “subchorionic placental cysts” [1,2,3].

SPCs are located beneath the chorionic plate, and their occurrence has been reported at various sites within or on the surface of the placenta. Commonly reported sites include the placental cord insertion site (PCIS), the area just beneath the fetal plate, and along the placental surface. In some cases, cysts have also been found within the placental septa or embedded in the placental tissue itself [4].

Previous studies have revealed an association between SPCs and maternal floor infarction (MFI), which is characterized by fibrin deposition and increased extravillous trophoblast proliferation (formerly known as X cells) [2,5]. Several studies have analyzed the cystic fluid contents in an attempt to elucidate the underlying pathophysiologic mechanisms [5,6]. However, the etiology remains poorly understood, and the clinical implications of these cysts remain controversial. In addition, while some authors have reported associations with adverse pregnancy outcomes, others have described normal outcomes.

Herein, we report a case of SPC detected prenatally using ultrasonography, with fetal growth restriction (FGR) and abnormal umbilical artery Doppler. In addition, we conducted a literature review of the reported cases to investigate the clinical outcomes of SPCs and examine the clinical significance of prenatal ultrasonographic diagnosis.

## 2. Case Report

### 2.1. Clinical Presentation

**Figure 1:**
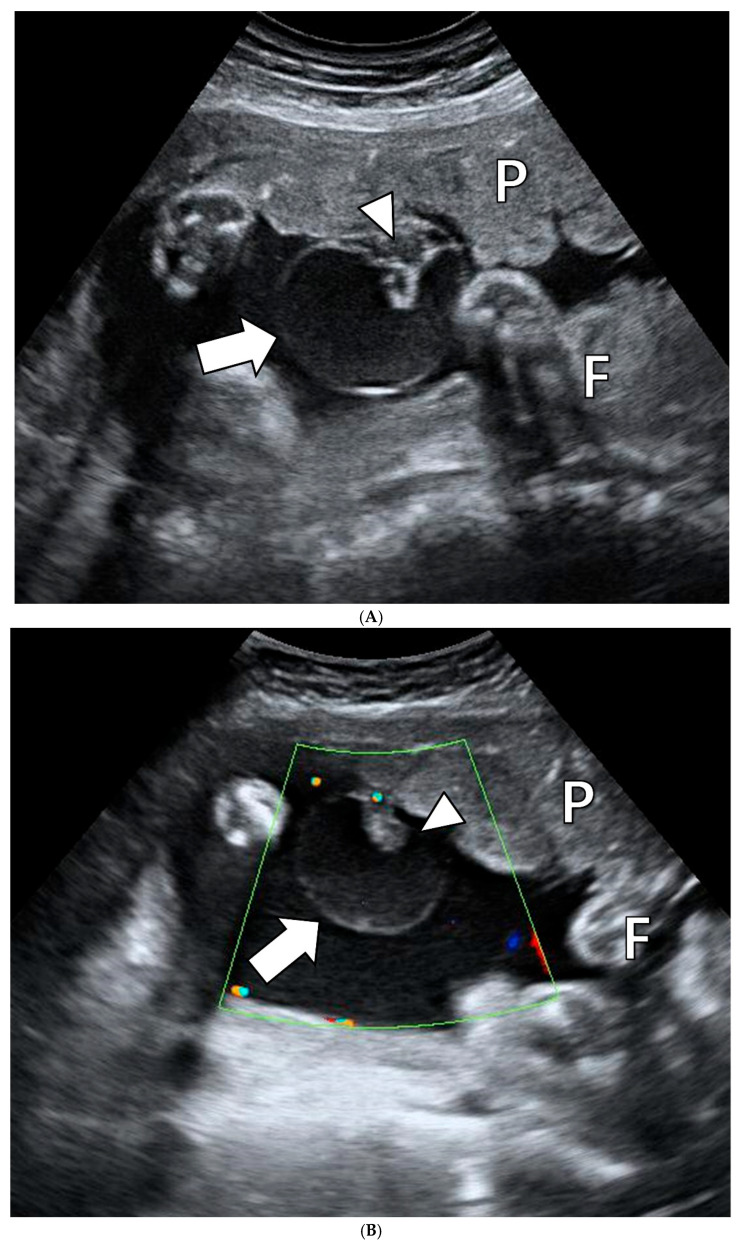
Prenatal ultrasonography of the subchorionic placental cyst at 35 weeks and 3 days of gestation. (**A**), An echolucent cystic mass is protruding from the fetal surface of the placenta (thick arrow). An intracystic heterogeneous echogenic lesion is shown (arrowhead). (**B**), Color Doppler images reveal no blood flow in the cyst. The cyst (thick arrow) and intracystic heterogeneous echogenic lesion (arrowhead). P, placenta; F, fetus.

A 40-year-old woman, gravida 7, para 0, presented to our department ty 28^3/7^ weeks of gestation due to asymmetric FGR and abnormal umbilical artery Doppler. She had a previous history of six spontaneous abortions and no underlying medical condition. The results of laboratory tests, including complete blood count, liver function, renal function, and coagulation tests, and C-reactive protein levels, were normal. There were no symptoms indicative of preeclampsia, and urine protein test results were negative; yet, she was diagnosed with gestational hypertension because of intermittently high blood pressure (BP) (systolic BP up to 150 mm Hg). Ultrasonography revealed that the estimated fetal weight (EFW) was 905 g (under the 3rd percentile). The FGR was asymmetric, with biparietal diameter at the 46.8th percentile, abdominal circumference under the 2.3rd percentile, and femur length under the 2.3rd percentile. Umbilical artery Doppler showed decreased end-diastolic flow, with increased pulsatility index (2.01), resistance index (0.99), and systolic to diastolic (S/D) ratio. All other sonographic findings, including amniotic fluid, fetal structures, cervical length, and placenta, were normal. In addition, cardiotocography (CTG) revealed threatened preterm labor, while fetal heart rate variability was normal. The patient was admitted to our department for tocolysis, and close observation was conducted. Ritodrine was used as a tocolytic agent, along with intramuscular dexamethasone and prophylactic antibiotics. Serial ultrasonography was conducted, which revealed improvement in the umbilical artery flow compared to that during the initial examination. At 28^6/7^ weeks of gestation, the umbilical artery S/D ratio decreased from 117.09 to 4.05 and thereby remained stable between 4 and 5 until delivery. Follow-up ultrasonography at 35^3/7^ weeks of gestation showed persistent FGR with no increase in EFW for a week and a newly detected cystic mass protruding from the fetal surface of the placenta. Sonographic findings were as follows (Figure 1): the cyst appeared independent from the fetus; was approximately 4 cm in diameter and located near the PCIS; was filled with echolucent fluid, which had an echodensity equivalent to the amniotic fluid; appeared as a 2 cm intracystic heterogeneous echogenic lesion compatible with clotting; and had a hyperechoic thin wall. Color Doppler imaging showed no blood flow inside the cyst. During the last 2 weeks, there was no increase in EFW, and CTG detected intermittent minimal variability and variable deceleration. Because of these findings, a cesarean section was scheduled at 36 weeks and 6 days of gestation, and a 1240 g (under the 1st percentile for gestational age [GA]) female baby was delivered with Apgar scores of 7 and 8 at 1 and 5 min, respectively. The baby was admitted to the neonatal intensive care unit and was examined and cared for as a preterm neonate. Although the baby was small for GA, no abnormalities were observed after a variety of tests, including a chromosomal abnormality screening test, brain and kidney ultrasonography, and echocardiography. Moreover, no infection was suspected, including routine screening for toxoplasmosis, rubella, cytomegalovirus, and herpes simplex virus. The baby was discharged without any problems after 33 days of hospitalization.

### 2.2. Pathologic Findings

**Figure 2:**
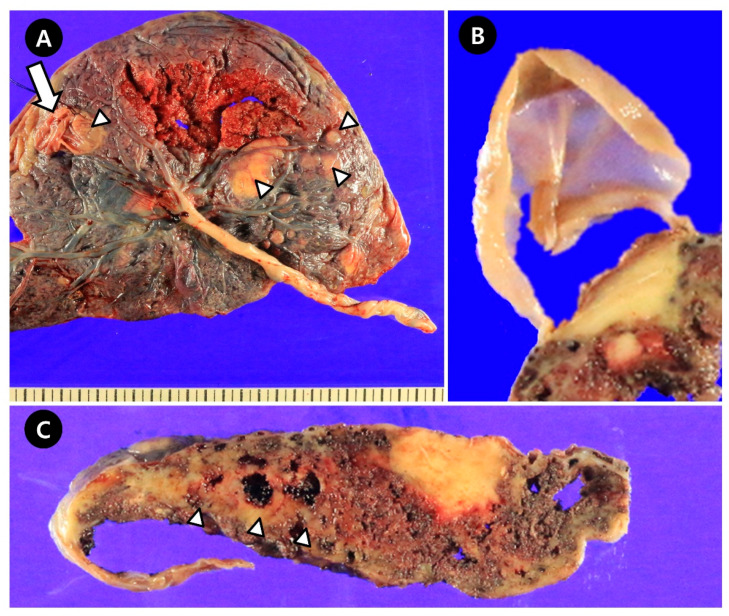
Gross photos of the placenta. (**A**), Fetal side of the placenta. A subchorionic placental cyst is observed on the fetal placental surface (thick arrow). Multifocal large waxy plaques are identified under the chorionic plate (arrowhead). (**B**), The largest ruptured cyst and the thin and translucent wall of the cyst. (**C**), Except for waxy plaques, multifocal waxy lesions that appear to be fibrin deposition are observed (arrowhead).

**Figure 3:**
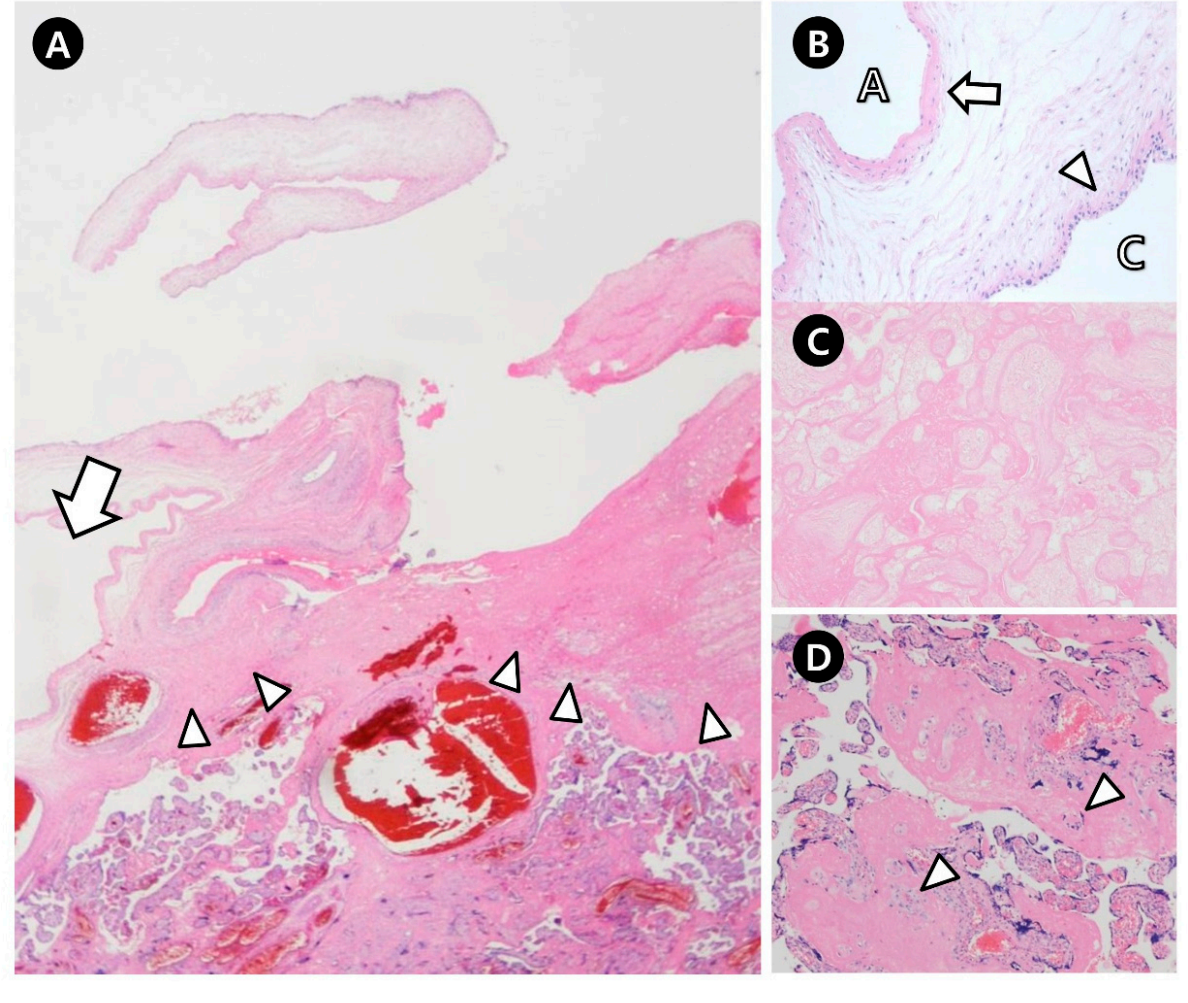
Microscopic photos of the placenta. (**A**), Subchorionic placental cyst (thick arrow) and the underlying waxy plaque (arrowhead). (**B**), The wall of the subchorionic placental cyst is composed of amnion (thick arrow) and chorion (arrowhead). (**C**), Placental infarction. (**D**), Pink colored perivillous fibrin depositions and extravillous trophoblasts (arrowhead). A, amniotic cavity; C, cyst cavity.

The weight of the placenta was 450 g, and the gross findings were as follows (Figure 2): the placenta appeared smaller than expected for gestational age; white to tan-colored nodular lesions, suspected to be fibrin deposits, were present; multiple bullae-like cysts were observed on the fetal surface—the largest one (4.1 × 3.2 cm) was ruptured, likely during delivery, and corresponded to the cyst previously identified on prenatal ultrasonography. The cyst had a thin, translucent wall. Microscopically, the lesion was confirmed to be an SPC overlying a waxy plaque of fibrin deposition, with a wall composed of amnion and chorion. Multiple placental infarctions (up to 4.2 × 2.0 cm) and multifocal perivillous fibrin deposits with increased extravillous trophoblasts (X-cells) were diffusely observed throughout the placenta, particularly around the cyst (Figure 3). However, no vasculopathy or thrombosis was observed.

Postoperatively, the mother’s blood pressure (BP) remained intermittently elevated, with systolic and diastolic pressures reaching up to 160/110 mmHg, although urinary protein remained negative and no other signs of preeclampsia were observed. Intermittent hypertension was effectively controlled with intravenous hydralazine. The patient was discharged on postoperative day 4 without complications, with her BP stabilized within the normal range.

## 3. Discussion

Few cases of SPCs have been reported in the literature. In the present study, we conducted a comprehensive review of cases reported up to August 2025. A systematic search was performed using PubMed with the following keywords: subchorionic cyst, placental cyst, chorionic cyst, membranous cyst, and subamniotic cyst. To ensure a thorough evaluation, only full-text articles published in English were included. To maintain diagnostic accuracy and allow for detailed pathological assessment, only cases with histopathologically confirmed SPCs were selected. Specifically, we included only those reports that provided detailed pathological descriptions regarding the size, number, and location of the cysts, as well as any specific histological findings and placental weight. Furthermore, studies lacking essential clinical information were excluded. Inclusion criteria required that each case report contain gestational age at diagnosis, estimated fetal weight, mode of delivery, birth weight, and perinatal outcomes. As a result, 16 cases of SCPCs (including the present case) were identified, and their clinicopathologic characteristics and obstetric outcomes were analyzed.

### 3.1. Patient Characteristics and Ultrasonographic Findings of SPCs

The median age of the patients was 33 years (range: 22–40), and seven women (47%) were >35 years. The cysts were found using prepartum ultrasonography in all but one case, with two (12.5%) detected in the first trimester, seven (43.8%) in the second trimester, and six (37.5%) in the third trimester (Table 1). Adverse outcomes in previous pregnancies were reported in three cases (cases 3, 12, 16) (Table 2).

Most reports have described ultrasonographic findings of cysts as showing echodensity similar to that of amniotic fluid, thin hyperechoic walls, and no intracystic blood flow on color Doppler. Additionally, intracystic hyperechoic lesions suggestive of hemorrhage or hematoma were observed in five cases (cases 9, 12, 13, 15, 16), and intracystic passage of the umbilical cord was observed in three cases (cases 1, 2, 10).

### 3.2. Differential Diagnosis of SPCs

If a placental cyst is detected on prenatal ultrasonography, the differential diagnosis includes subchorionic or subamniotic thrombohematoma, chorioangioma, and placental avillous spaces (placental lakes) [12]. Thrombohematomas and chorioangiomas typically appear as heterogeneous echodense masses [10]. Chorioangiomas can be distinguished by the presence of color Doppler flow within the lesion. Placental lakes, defined as anechoic areas measuring at least 1 cm in their largest diameter and demonstrating turbulent blood flow on real-time ultrasonography, are not associated with adverse pregnancy outcomes [17].

A few studies have reported that elevated maternal serum alpha-fetoprotein (AFP) levels are associated with placental abnormalities such as subchorionic hemorrhage, sonolucent areas, and multiple cystic lesions occupying the placenta, even when fetal anatomy appears normal on ultrasound [18]. However, no study to date has specifically reported an association between SPCs and elevated maternal serum AFP levels.

### 3.3. Etiology of SPCs

Several studies have suggested that extravillous trophoblast cells may be involved in the formation of SPCs [4,5,9]. Although the precise functions of these cells remain unclear, some evidence supports their secretory activity as a potential source of the cystic fluid [4]. Furthermore, emerging data indicate a possible association between the occurrence of SPCs and subamniotic hemorrhage. A recent case report provided compelling evidence that fluid aspirated from placental cysts in fetuses affected by FGR and anemia contained blood of fetal origin [15].

### 3.4. Placental Hypoxia and Infarction in Cases with SPCs

The placental cyst has been reported to influence placental hypoxia and maternal floor infarction (MFI) [2,5,13]. However, the exact pathogenetic mechanism is currently unclear. SPCs typically form in the subchorionic region, which lies anatomically above the intervillous space. Although SPCs do not originate from the intervillous space itself, large cysts may compress or displace this space, potentially impairing maternal–fetal exchange [12]. Placental hypoxia, which is related to abnormal placentation in early gestation and placental vascular disease in late gestation, is known to be a common finding in pregnancies complicated by intrauterine growth restriction, gestational hypertension, or preeclampsia [19,20]. MFI, defined as massive perivillous fibrin deposition, represents a dense fibrinoid layer extending beyond the basal plate, suffocating the villi and obliterating the intervillous space. Consequently, gas and nutrient exchange across the affected villi are impaired. Perivillous fibrin deposition is associated with recurrent adverse pregnancy outcomes, including miscarriage, FGR, preterm delivery, and stillbirth [20,21].

Among the findings suggestive of placental hypoxia, the following were detected in the present case: fibrin deposition, MFI, increased extravillous trophoblast cells, and small placental weight. Among the 16 reviewed cases, fibrin deposition was reported in nine cases (56.3%), MFI or simply described as placental infarction in five cases (31.3%), and increased extravillous trophoblast cells in eight cases (50%). Three cases did not have any other pathologic abnormality of the placenta, except for SPCs. These three cases were reported in 1986 and 1996; therefore, there may have been missing information in the pathologic findings, in particular, from earlier studies performed when pathologic investigations were limited. Close examination of the cord and placental vessels was conducted in the present case, but no vasculopathy was detected, suggesting that placental hypoxia was not caused by vasculopathy such as thrombosis or vascular occlusion. In the literature review, there was a case of SPCs accompanied by a single umbilical artery, along with subamniotic hemorrhage and placental infarction [15].

### 3.5. Relationship Between the Size, Number, and Location of SPCs and Adverse Pregnancy Outcomes

The most common SPCs are usually smaller than 2 cm in diameter and are innocuous [3]. Accordingly, small (<2 cm), isolated SPCs are reported not to require any changes in clinical management, and routine ultrasound follow-up is generally sufficient [2].

Among the 16 cases we reviewed, based on pathological findings, the cyst size was larger than 4.5 cm in 13 cases (81.3%), and the number of cysts was three or more in four cases (25%) (Table 1). Interestingly, neither larger cyst size nor a higher number of SPCs appeared to increase the risk of adverse pregnancy outcomes. All three cases with SPCs smaller than 4.5 cm, as well as six of twelve cases with only one or two SPCs, experienced at least one adverse pregnancy outcome (Table 2). Additionally, an increase in cyst size as the pregnancy progressed was observed in six cases (cases 4, 6, 7, 9, 11, and 12); however, this did not appear to have a significant impact on pregnancy outcomes either.

Brown et al. [4], in a retrospective series of 34 cases, reported that if placental cystic lesions are larger than 4.5 cm or if there are three or more cysts, FGR occurs more frequently. This report has been frequently cited by subsequent studies on SPCs; however, it was not limited to pathologically confirmed SPCs but included a variety of placental surface cysts detected on prenatal ultrasound. As a result, it encompassed various pathological entities, such as amniotic cysts, placental hematomas, and pseudocysts. Only a portion of the cases underwent pathological examination, and in several cases, no cyst was identified upon pathological review. Therefore, clinical outcomes specific to SPCs could not be evaluated based on this study.

Some authors have suggested that the location of the cyst near the PCIS may cause umbilical cord displacement or constriction, reducing umbilical cord blood flow and thus leading to FGR [3,7,8]. However, the location near the PCIS is probably not a predictive factor for FGR. In our review of the literature, all cases were observed near the PCIS, except for two with missing information about the cyst location.

### 3.6. Placental Weight in Cases with SPCs

A small placental weight is known to be associated with a range of pathologies, including FGR and preeclampsia, but there is also evidence of an association with placental hypoxia [22]. We calculated the percentiles of placental weight for GA according to the placenta weight percentile curves reported by Almog et al. [16]. Among the 16 reviewed cases, eight (50%) were below the 10th percentile—including three cases (18.8%) below the 3rd percentile and five (31.3%) between the 3rd and 10th percentiles. Additionally, two cases (12.5%) fell between the 10th and 25th percentiles, another four (25%) between the 25th and 50th percentiles, and two cases (12.5%) were above the 50th percentile (Table 1). In our literature review, five cases (cases 3, 4, 7, 15, 16) presented with small birth weight for GA; among these, placental weight was below the 10th percentile in three. None of the studies reported the occurrence of preeclampsia, although one case presented with pregnancy-induced hypertension (case 2), where the placental weight was under the 3rd percentile, but the birth weight of the neonate was in the 40th percentile. Accordingly, a significant proportion of SPCs are associated with low placental weight, which appears to be related to low birth weight.

### 3.7. Adverse Pregnancy Outcomes in Cases with SPCs

As mentioned above, the clinical significance of cysts has been poorly evaluated. Our results from reviewing pregnancy outcomes for 16 cases of SPCs are as follows: FGR, defined as fetal weight below the 10th percentile for GA using ultrasonography, was reported in 8 cases (50%); abnormal color Doppler findings of the middle cerebral artery or umbilical vessels were reported in four cases (25%); preterm labor was reported in three cases (18.8%); and preterm birth occurred in eight cases (80%), of which three cases (18.8%) were delivered before 34 weeks of gestation (Table 2). A cesarean section was performed in 12 cases (75%); excluding three cases of repeat cesarean sections, the reasons for cesarean delivery varied, including FGR, abnormal CTG, transverse lie presentation due to the large SPC, and oligohydramnios. Several reasons have been described for considering cesarean delivery even in the absence of abnormalities, including a high risk of cyst rupture during labor, potential placental bleeding upon rupture, and the risk of fetal aspiration of intracystic fluid [12]. Considering these results, SPCs may tend to be associated with unfavorable pregnancy outcomes such as FGR, preterm birth, and an increased risk of cesarean section.

### 3.8. Study Limitations

There are several limitations in our review of the literature. First, this report inevitably involves a selection bias because only published cases were reviewed. Because only complicated cases tend to be reported, cases of SPC with normal outcomes may have been missed. Second, neonatal complications were not reported in any of the 16 cases, except for one case of neonatal anemia; however, detailed neonatal evaluations and follow-up data were not provided. Although the newborn was discharged without complications, long-term follow-up data are needed to assess potential complications, including neurodevelopmental delays, intellectual disabilities, cognitive impairments, and other related outcomes. Third, the number of cases was too small to evaluate statistical significance. Lastly, among the reviewed cases, genetic evaluation was conducted only in a few, and even then, only through amniocentesis or neonatal gene tests; genetic analysis of the placenta or the mother was not conducted in any case. Several studies have reported that placental dysfunction in preeclampsia is associated with single-nucleotide polymorphisms [23,24]. Future studies are needed to examine whether single-nucleotide polymorphisms are relevant in placental hypoxia, which is associated with SPCs.

## 4. Conclusions

SPCs appear to be associated with unfavorable pregnancy outcomes; however, they do not seem to act as independent causes of obstetrical complications. By focusing on their association with placental hypoxia, we propose that SPCs could potentially serve as clinical markers of placental hypoxia. Nevertheless, based on the current review, it remains difficult to determine whether SPCs are a causal factor, a consequence, or merely an epiphenomenon.

Placental hypoxia is already known to influence obstetric complications; however, none of its hallmark findings, such as fibrin deposition or MFI, can be detected using prenatal ultrasonography. Therefore, if SPCs are detected, strict management—including serial CTG and ultrasound examinations to monitor FGR—may be required to determine the most appropriate timing and method of delivery. A hospital equipped with a neonatal intensive care unit for the care of preterm infants is, however, also necessary. Furthermore, placental hypoxia and its associated adverse pregnancy outcomes can recur in subsequent pregnancies; therefore, obstetricians who detect SPCs should recommend pathological examination of the placenta to assess for placental hypoxia. Future studies with larger sample sizes and longer follow-up periods are warranted to better understand the etiopathology and to develop possible preventive interventions.

## Figures and Tables

**Table 1:** Review of the literature on the clinicopathologic characteristics of patients with subchorionic placental cysts.

Case No.	Age (Years)	GA at Diagnosis (Weeks)	Cyst Size (cm) ^1^	Number of Cysts ^2^	Weight of the Placenta (g) ^3^
1 [7]	39	30	5	1	530
2 [7]	38	32	8	1	370 ^4^
3 [5]	29	Postpartum	4	Multiple (>5)	385 ^4^
4 [8]	28	12	7	1	480 ^4^
5 [9]	31	36	6	1	435 ^4^
6 [9]	22	16	12	1	450 ^4^
7 [10]	35	16	8	1	390 ^4^
8 [11]	31	26	4.6	1	619
9 [12]	35	<31	7.2	1	450 ^4^
10 [13]	36	24	3	Multiple (>3)	320
11 [1]	29	7^2/7^	20	1	383 ^4^
12 [14]	29	26	7.7	5	592
13 [2]	37	26	6	2	513
14 [3]	33	28	9	1	800
15 [15]	NA	22	5.8	Multiple (>5)	330
16 (present case)	40	35^3/7^	4.1	1	421 ^4^

^1,2^ Number and size (the longest length) of the cysts by pathologic examination. If no pathologic information exists, results were substituted as ultrasound findings. ^3^ Placenta weight by pathologic examination. ^4^ Less than 10 percentiles of placental weight for GA. Placenta weight percentile for GA was determined based on the reference curves published by Almog et al. in 2011 [16]. GA, gestational age; NA, not available.

**Table 2:** Review of the literature on the obstetric outcomes of patients with subchorionic placental cysts.

Case No.	GA at Delivery (Weeks)	FGR ^1^	Delivery Mode	Birth Weight (g, Percentile) ^2^	Adverse Obstetrical Findings
1 [7]	37^6/7^	Yes	VD	2540 (9–14)	Abnormal Doppler ultrasound: Absent end diastolic velocity in UA and in the fetal descending aorta, and reduced velocity in the fetal umbilical vein, Preterm labor
2 [7]	39	No	VD	3250 (40)	PIH
3 [5]	34^6/7^	Yes	CS	1325 (<1)	Previous cesarean section due to fetal distress with FGR and placenta previa, Non-reassuring fetal status
4 [8]	37	Yes	CS	2100 (4)	Non-reassuring fetal status, Abnormal Doppler ultrasound: Low impedance in MCA (RI: 0.61), higher impedance in UA (RI: 0.78), and decreased umbilical/cerebral index (0.88)
5 [9]	39^5/7^	No	VD	3250 (29)	None
6 [9]	39	No	CS	2950 (27)	None
7 [10]	34	Yes	CS	1290 (1)	Oligohydramnios, Amniocentesis: 45XY, t(13q,14q)
8 [11]	>37	No	VD	3660 (95)	None
9 [12]	37	No	CS	3330 (85)	Non-reassuring fetal status
10 [13]	29	No	CS	1092 (41)	Non-reassuring fetal status, Short cervix (2 cm)
11 [1]	36	No	CS	2585 (48)	None
12 [14]	32	Yes	CS	1389 (21)	Previous one spontaneous abortion, and one CS due to IUFD caused by placental abruption, Preterm labor, Placental abruption
13 [2]	34	Yes	CS	1850 (25)	None
14 [3]	37	No	CS	2950 (60)	None
15 [15]	29^4/7^	Yes	CS	730 (5%)	Non-reassuring fetal status, Abnormal Doppler ultrasound: A reversed A-wave in the ductus venosus, an elevated peak systolic velocity of MCA (1.58 multiples of the median), and persistent reversed end-diastolic velocities in UA, Single umbilical artery, Neonatal anemia
16 (present case)	36^6/7^	Yes	CS	1240 (<1)	6 previous spontaneous abortions, Non-reassuring fetal status, Abnormal Doppler ultrasound: Increased PI in UA (PI: 2.01), RI in UA (0.99), Decreased S/D ratio (117.09), Preterm labor

^1^ FGR is defined as sonographically estimated fetal weight under the 10th percentile for gestational age. ^2^ Percentile of infant birth weight calculated by Fenton 2013 Growth Calculator. GA, gestational age; FGR, fetal growth restriction; VD, vaginal delivery; CS, cesarean section; UA, umbilical artery; PIH, pregnancy-induced hypertension; MCA, middle cerebral artery; RI, resistance index; IUFD, intrauterine fetal death; PI, pulsatility index; S/D ratio, systolic/diastolic ratio.

## Data Availability

The original contributions presented in the study are included in the article. Further inquiries can be directed to the corresponding author.
